# Highly defective graphene quantum dots-doped 1T/2H-MoS_2_ as an efficient composite catalyst for the hydrogen evolution reaction

**DOI:** 10.1038/s41598-023-42410-9

**Published:** 2023-09-13

**Authors:** Sheng-Fu Chen, Tai‑Sing Wu, Yun‑Liang Soo

**Affiliations:** 1 Department of Physics, National Tsing Hua University, Hsinchu, Taiwan; 2 National Synchrotron Radiation Research Center, Hsinchu, Taiwan

**Keywords:** Catalysis, Materials for energy and catalysis, Chemical physics

## Abstract

We present a new composite catalyst system of highly defective graphene quantum dots (HDGQDs)-doped 1T/2H-MoS_2_ for efficient hydrogen evolution reactions (HER). The high electrocatalytic activity, represented by an overpotential of 136.9 mV and a Tafel slope of 57.1 mV/decade, is due to improved conductivity, a larger number of active sites in 1T-MoS_2_ compared to that in 2H-MoS_2_, and additional defects introduced by HDGQDs. High-resolution transmission electron microscopy (HRTEM), Raman spectroscopy, x-ray diffraction (XRD) and x-ray photoelectron spectroscopy (XPS) were used to characterize both the 1T/2H-MoS_2_ and GQDs components while Fourier-transform infrared spectroscopy (FTIR) was employed to identify the functional groups on the edge and defect sites in the HDGQDs. The morphology of the composite catalyst was also examined by field emission scanning electron microscopy (FESEM). All experimental data demonstrated that each component contributes unique advantages that synergistically lead to the significantly improved electrocatalytic activity for HER in the composite catalyst system.

## Introduction

Electrocatalytic hydrolysis is considered as one of the most environmentally friendly methods for hydrogen production due to its non-polluting and recyclable nature. In search of high-performance and low-cost catalysts for electrochemical hydrogen evolution reactions (HER), transition metal dichalcogenides have been noted to be favorable candidates owing to their unique two-dimensional properties that can improve catalytic capability. In the present work, we are especially interested in the MoS_2_ system, which has been demonstrated to have excellent HER catalytic activity and low-onset overpotential.^1,2^ The electrical conductivity and the number of active sites from sulfur vacancies, which are on the edges in 2H- and 1T-MoS_2_^3^ and on the basal plane in 1T-MoS_2_^4^, are the two main factors that determine the catalytic efficiency of the material. The most stable form, 2H-MoS_2,_ is a semiconductor with low carrier transport efficiency and poor electrical conductivity that restrict its catalytic performance.^5^ To improve electrical conductivity, many studies have focused on using the metallic 1T phase to replace the semiconducting 2H phase of MoS_2_.^6–9^ However, the 1T phase of MoS_2_ is unstable and can easily transition to the 2H phase. Alternatively, the catalytic performance of MoS_2_ may be improved by decorating its active sites. In particular, graphene quantum dots (GQDs) are used to introduce numerous defects on the active sites of MoS_2_, enhancing the electrical conductivity and catalytic activity.^10–12^ However, the hydrothermal method, usually used for incorporating GQDs into MoS_2_, can easily induce 1T-to-2H phase transition in the MoS_2_ host due to the elevated temperature above 190℃ used in the process.^10^ Therefore, recent studies on GQD-doped MoS_2_ have been mainly based on the 2H phase.^10,13^.

In this paper, we present an easy method to prepare a new composite catalyst system: a combination of a carbon paper as the substrate, highly defective GQDs (HDGQDs) as an enhancer, and 1T/2H-MoS_2_ as the main catalytic agent. The 1T/2H-MoS_2_ was prepared by a Li-ion intercalation process, while the HDGQDs were fabricated from citric acid using the pyrolysis carbonization method. The HDGQDs and 1T/2H-MoS_2_ solutions were then dropped and air-dried on the carbon paper to form the composite catalyst system. We note that no additional heating was required to combine the HDGQDs with MoS_2_ and the 1T phase of MoS_2_ was successfully preserved. The three goals of improving electrical conductivity, increasing the number of active sites, and enhancing the catalytic performance of the active sites are therefore simultaneously accomplished. Our 1T/2H-MoS_2_/HDGQDs catalyst exhibits excellent HER electrocatalytic performance. This work provides a unique new strategy for the preparation of high-performance water electrolysis catalysts.

## Methods

The 1T/2H-MoS_2_/HDGQDs composite catalyst system was fabricated by sequentially dropping the HDGQDs (0.2 ml, 6 mg/ml) or GQDs (0.0545 ml, 22 mg/ml)^14^ and 1T/2H-MoS_2_ solutions (0.2 ml) onto the carbon paper substrate (CeTech, GPP035), covering a circular area of radius 0.3 cm, and then letting them air dry. The dosages for 1T/2H-MoS_2_ and HDGQDs were tested to achieve optimal loading, with further details provided in the supplementary information. As shown in Supplementary Figure S1, S2 and Table S1, we determined that 0.2 ml of MoS_2_ is the optimal loading on the Carbon paper as the primary catalytic agent. When the MoS_2_ loading exceeds 0.2 ml, the efficiency decreases due to increased resistance, which hinders the hydrogen evolution reaction. As depicted in Supplementary Figure S3, we found that 0.2 ml of HDGQDs is the optimal loading for enhancing the MoS_2_ catalyst. Further addition of HDGQDs does not increase efficiency, as the active sites of MoS_2_ are already saturated.

To prepare the HDGQDs solution, citric acid powders (2 g) in a glass flask were oil-bath heated to 200℃ for 30 min. The orange liquid of pyrolyzed citric acid was mixed with deionized water (20 ml) to form a HDGQDs solution, which was then purified by filtering using a 0.22 μm microporous membrane and dialyzed using a cellulose ester membrane bag (flat width 54 mm, diameter 34 mm, MWCO 3.5 KD) for two days, with several water changes during the dialysis process. For comparison, we also fabricated standard GQDs by increasing the heating time from 30 to 90 min in the above process.

The 1T/2H-MoS_2_ solution was prepared by using an improved lithium ion intercalation-based exfoliation process. Molybdenum (IV) sulfide 99% powders of 90 nm-diameter (0.1 g) and n-Butyl lithium (5 ml) were mixed in an autoclave reactor and heated to 110℃ in an oven for 12 h. In contrast to the previous method, where 0.5 g bulk MoS_2_ powder was soaked in 4 ml of 1.6 M n-butyllithium/hexane for 48 hours^15^, this approach can significantly enhance the efficiency of lithium ion insertion. After cooling down to room temperature, the mixture was diluted in n-hexane and centrifuged to remove excessive n-Butyl lithium not intercalated with MoS_2_, leaving the Li_x_MoS_2_ as precipitates. Hydrochloric acid solution (150 ml, pH = 3) was then added to exfoliate MoS_2_ few layer sheets from the Li_x_MoS_2_ precipitates. Finally, the MoS_2_ solution was purified by centrifugation, filtration (pore size 0.22 μm, CA membrane filter) and dialysis (Peristaltic pump rapid dialysis device, MWCO 12–14 kD hollow fiber) to obtain a neutral 1T/2H-MoS_2_ solution. Compared to the previous method, where hydrochloric acid solution was dropwise added to an alkaline MoS_2_ colloidal solution containing trace amounts of lithium hydroxide until the pH reached around 7^15^, this milder dialysis approach avoids residual chloride ions and exothermic acid–base reactions. This prevents phase change and oxidation of MoS_2_. On the other hand, the milder dialysis approach can effectively remove LiOH and concentrate the sample according to experimental requirements. For the Raman and XPS measurements, the solution was dropped on a carbon paper substrate and air dried to form the 1T/2H-MoS_2_ sample. A more thermally stable 2H-MoS_2_ sample was also prepared by heating the 1T/2H-MoS_2_ sample at 300℃ for 1 h in vacuum^16^.

To characterize the GQDs and 1T/2H-MoS_2_ components of the composite catalysts, a high-resolution transmission electron microscope (HR-TEM) (JEOL JEM-2010) with acceleration voltage 200 kV was used to probe the microstructures. Raman spectra were obtained using a Raman spectrometer with a CCD camera (HORIBA, iHR-550) and a 514 nm excitation laser. The Raman band for Si at ~ 520.7 cm^−1^ was used as a reference to calibrate the spectrometer. The x-ray diffraction (XRD) patterns in the 2*θ* range of 5–40° were recorded on an in-house x-ray diffractometer (Bruker, D8 discover plus) using the Cu-Kα radiation (*λ* = 0.15418 nm). Fourier-transform infrared spectroscopy (FTIR) was also obtained, by using an FT-IR spectrophotometer (Bruker, Vertex80v), to identify the functional groups on the edge and defect sites in the HDGQDs sample. The chemical environments surrounding C and O in GQDs and Mo and S in MoS_2_ were investigated by x-ray photoelectron spectroscopy (ULVAC-PHI, PHI Quantera II, with binding energies corrected using the C 1 s peak of 284.5 eV). Finally, the morphologies of the composite catalyst systems were examined by field emission scanning electron microscopy (FE-SEM) (JEOL 7900F).

Electrochemical analyses including linear sweep voltammetry (LSV), Tafel plots, electrochemical impedance spectroscopy (EIS), and stability test were carried out to evaluate the electrocatalytic activity for the new composite catalyst system of 1T/2H-MoS_2_/HDGQDs on carbon paper substrate. An electrolytic cell with the electrocatalyst, an Ag/AgCl electrode, and a glassy carbon electrode as the working, reference, and counter electrodes, respectively, submerged in a 0.5 M H_2_SO_4_ electrolyte solution was connected to a CHI electrochemical workstation (CHI Instruments 760D) for all the electrochemical measurements. The LSV curves were scanned with a scan rate of 5 mV/s. The LSV curve of a blank carbon paper substrate was also measured to perform background subtraction and baseline correction for all samples^17^.A 1.5 Ω small series resistance is used to treat LSV curves for iR correction^18^. The electrochemical potentials in these measurements were converted to the reversible hydrogen electrode (RHE) scale^19^. Using the Tafel equation, Tafel plots were also obtained from the LSV data to calculate the Tafel slopes^20^. The EIS data were measured at an overpotential of − 0.3 V vs. RHE in the frequency range of 0.001 ~ 100 kHz with an amplitude of 5 mV^10,12^. Chronopotentiometry experiments were carried out in a 0.5 M H_2_SO_4_ solution with a constant current density of 10 mA/cm^2^.

## Results and discussion

**Figure 1:**
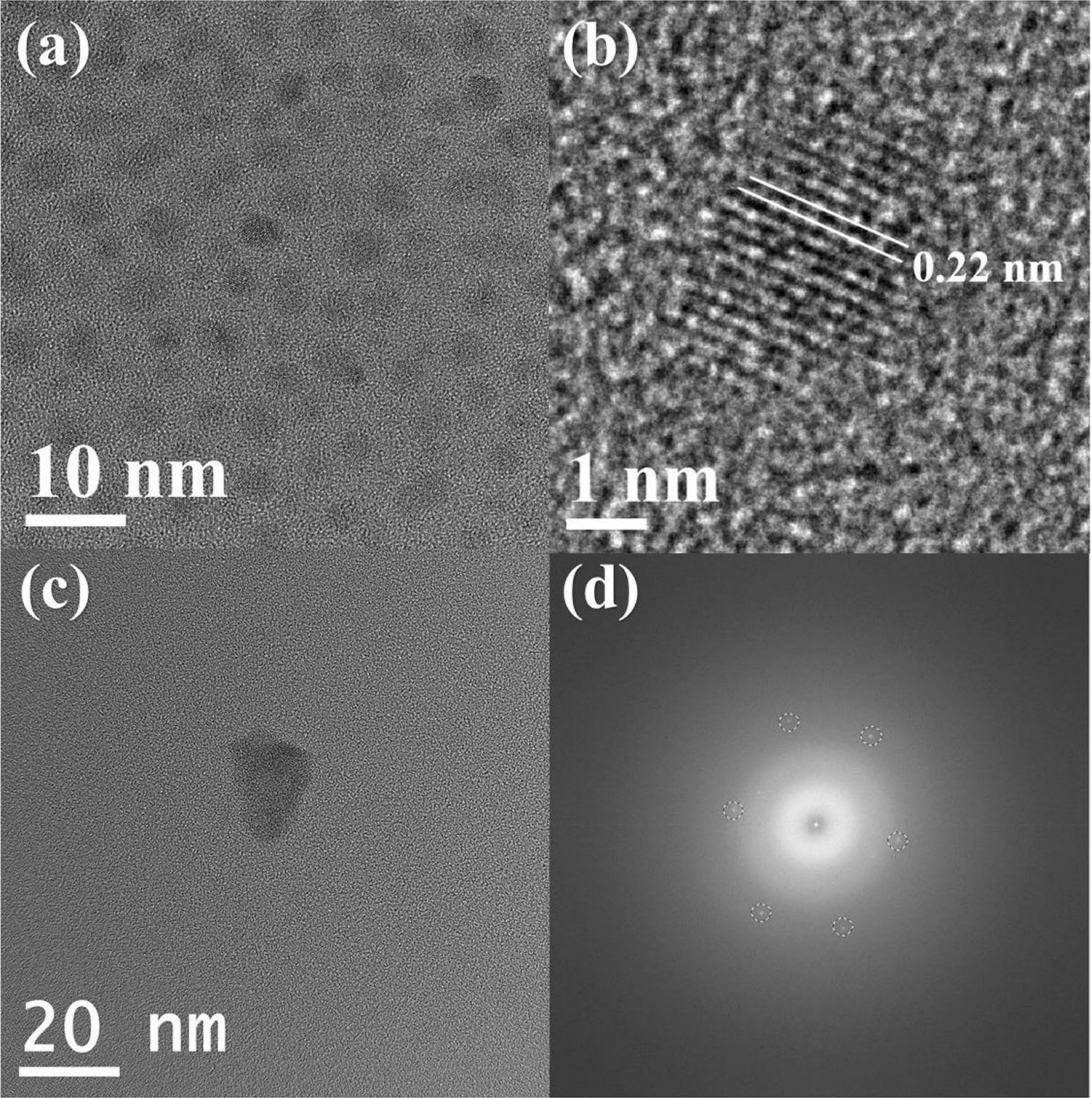
(**a**) TEM micrograph of HDGQDs. (**b**) HRTEM micrograph of HDGQDs. (**c**) TEM micrograph of standard GQDs. (**d**) FFT pattern of standard GQDs.

As demonstrated in Fig. 1a, the TEM micrograph shows that the particle size of the HDGQDs is around 3-5 nm. The graphite (1120) planes with a d-spacing of 0.22nm^21^ can also be clearly seen in the high-resolution TEM (HRTEM) micrograph (Fig. 1b). However, no clear pattern was observed in the fast Fourier transform of the micrograph, probably due to the material’s highly defective structure. In contrast, as shown in Fig. 1c, the TEM micrograph of the standard GQDs sample exhibits an appreciably larger particle size of 20 nm and its fast Fourier transform shows clear pattern of six-fold symmetry^22^ (Fig. 1d), indicating that the increased heating time of 90 min can effectively eliminate defects and enlarge graphene particles at the same time.

**Figure 2:**
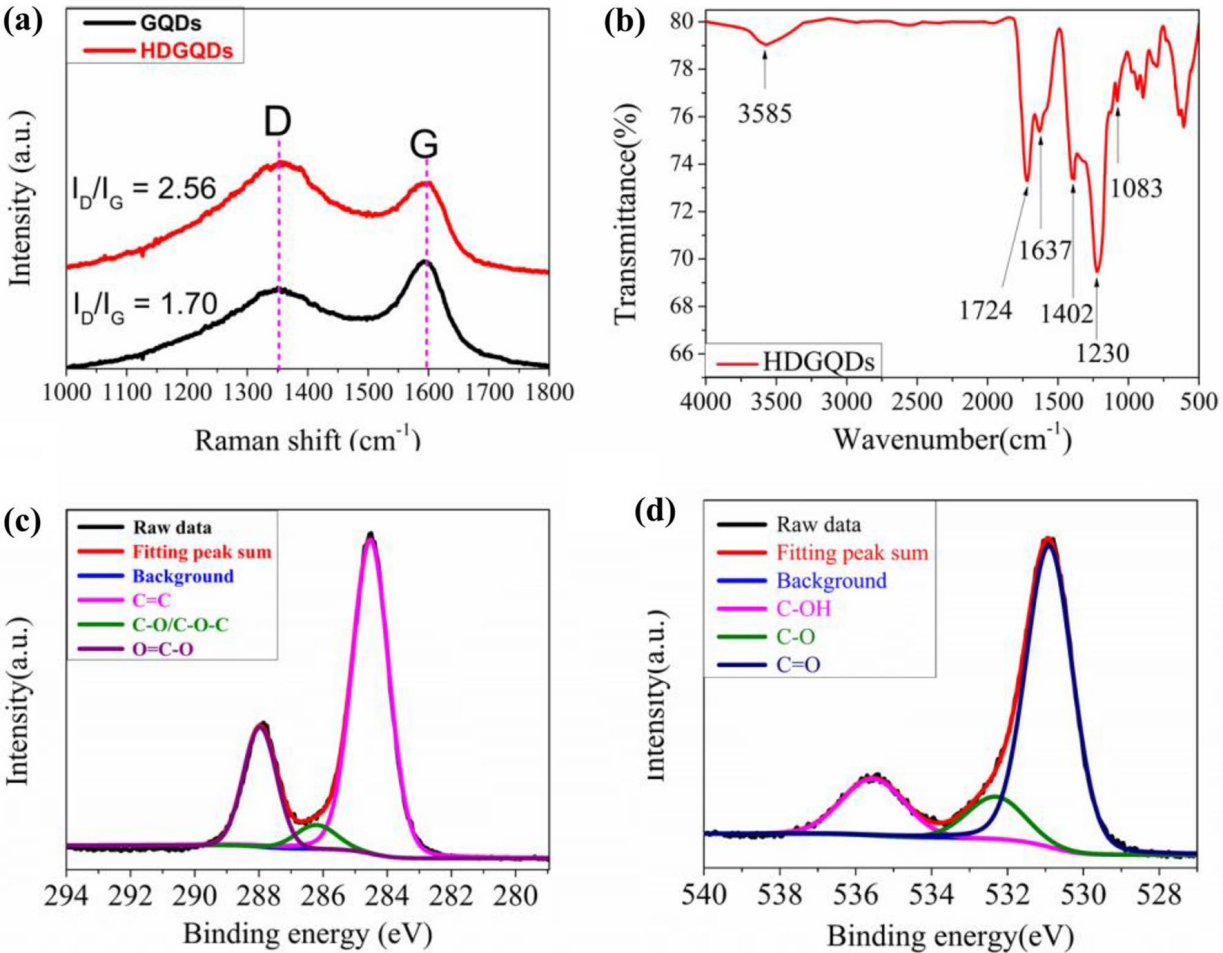
(**a**) Raman spectra of HDGQDs and GQDs. (**b**) FTIR spectrum of HDGQDs. (**c**) High-resolution C1s XPS spectra of HDGQDs. (**d**) High-resolution O1s XPS spectra of HDGQDs.

The Raman spectroscopy measurements can be used to compare the number of defects in the HDGQDs with that in the standard GQDs. As demonstrated in Fig. 2a, two peaks are present in the spectra of both the HDGQDs and standard GQD samples. The peaks at 1355 cm^−1^ and 1595 cm^−1^ are attributed to the D band and G band which represent the defect-related out-of-plane vibration and the in-plane sp^2^ mode of graphene, respectively. Therefore, the ratio of peak intensities of the D and G bands can be used to evaluate the defective structure of the sample^23^. The D/G ratio of 2.56 for the HDGQDs sample is appreciably higher than that of 1.70 for the standard GQDs sample. The Raman spectroscopy results demonstrate that the number of defects in GQDs samples fabricated from pyrolyzing citric acid can be controlled by adjusting the heating time.

As shown in Fig. 2b, peaks at 3585 cm^−1^, 1724 cm^−1^, 1083 cm^−1^, and 1402 cm^−1^, representing the stretching vibrations of O-H^14,21,24–26^, C = O^14,26^, C-O^21,24^, and in-plane bending vibration of O-H^14,21,24,25^ in the carboxyl groups, respectively, appear in the FTIR spectrum. A peak at 1637 cm^−1^ ascribed to skeletal ring vibrations of aromatic C = C^21,25^ in the graphitic domain and a peak at 1230 cm^−1^ attributed to stretching vibrations of C-O^14,24,25^ in the -COC bond, are also present.

X-ray photoelectron spectroscopy (XPS) was used to identify the chemical environments of carbon and oxygen atoms in the HDGQDs sample. As shown in Fig. 2c, three peaks centered at 288.5 eV, 286.3 eV and 284.6 eV assigned to O = C-O, C-O/C–O–C, and sp^2^ C = C bonding, respectively, were observed in the C1s spectrum^11,13,21,27–29^. In the O1s spectrum shown in Fig. 2d, the peaks at 535.6 eV, 532.3 and 530.9 eV are attributed to the C–O–H, C–O, and C = O bonding, respectively^27^. The chemical bonding of C and O revealed by XPS measurements is consistent with the presence of the functional groups observed from FTIR data in the HDGQDs samples (Fig. 2b).

**Figure 3:**
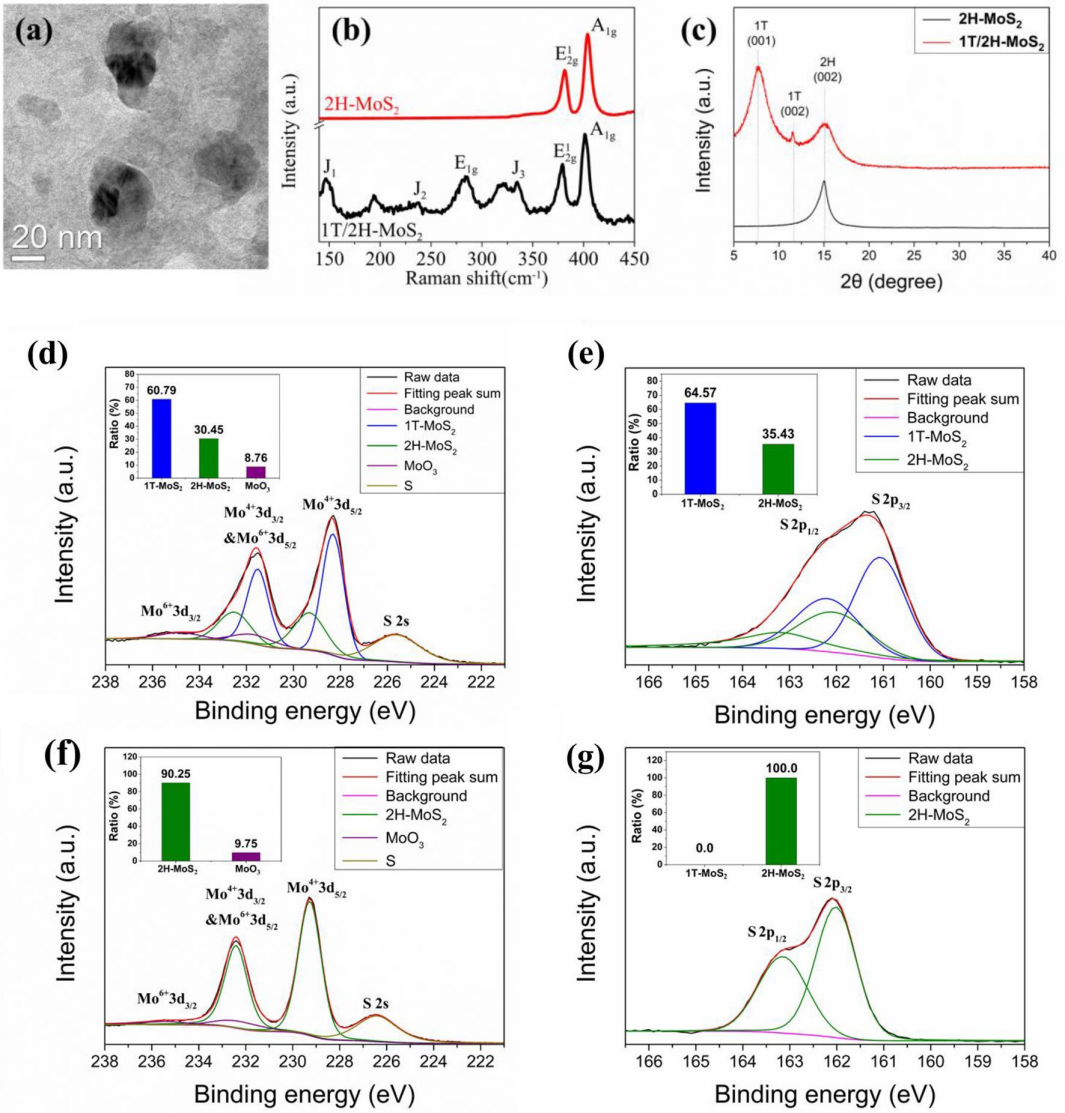
(**a**) TEM micrograph of 1T/2H-MoS_2_. (**b**) Raman spectra of 1T/2H-MoS_2_ and 2H-MoS_2_. and (**c**) XRD patterns of 1T/2H-MoS_2_ and 2H-MoS_2_. (**d**) XPS spectrum of Mo 3d for 1T/2H-MoS_2._ (**e**) XPS spectrum of S 2p for 1T/2H-MoS_2_. (**f**) XPS spectrum of Mo 3d for 2H-MoS_2_. (**g**) XPS spectrum of S 2p for 2H-MoS_2_.

As shown in Fig. 3a, the particle size of 1T/2H-MoS_2_ revealed by the TEM micrograph is around 20–40 nm, substantially larger than the HDGQDs size of 3–5 nm. The Raman data plotted in Fig. 3b shows that both the 1T/2H-MoS_2_ and 2H-MoS_2_ spectra have two major peaks: a peak at 379.4 or 381.5 cm^−1^ (E^1^_2g_), representing the vibration of two sulfur atoms with respect to molybdenum, and a peak at 401.0 or 4.03,8 cm^−1^ (A_1g_), representing the relative vibration of S atoms in the out of plane direction^8^. However, the 1T/2H-MoS_2_ spectrum exhibits four additional peaks at 146.8 cm^−1^(J_1_), 237.6 cm^−1^(J_2_), 283.7 cm^−1^(E_1g_), and 334.7 cm^−1^(J_3_)^6^. The E_1g_ mode is attributed to the octahedral coordination of Mo in 1T-MoS_2_^30^ while the J_1_, J_2_, and J_3_ modes are due to the superlattice structure of 1T-MoS_2_^31^. Therefore, the Raman results indicate that our 1T/2H-MoS_2_ sample indeed contains both the 1T-MoS_2_ and 2H-MoS_2_ phases.

Figure 3c shows the XRD patterns of the 1T/2H-MoS_2_ and 2H-MoS_2_ samples. A diffraction peak centered at around 15.0° corresponding to the (002) planes of the 2H-MoS_2_ structure appeared in the data of the 2H-MoS_2_ samples. On the other hand, three peaks at around 7.8°, 11.6° and 15.1° representing the 1T (001), 1T (002) and 2H (002) planes, respectively, were observed in the data of the 1T/2H-MoS_2_ sample^32–34^. According to the Scherrer equation, D = κλ/βcos(*θ*), where *κ* = 0.9 and *λ* = 0.154056 (nm), the grain size for the 2H-MoS_2_ sample with 2H (002) peak position 2*θ* = 15.0° and peak width *β* = 1.44° was estimated to be 5.6 nm. For the 1T/2H-MoS_2_ sample, the 1T-MoS_2_ grain size calculated from the 1T(001) peak at 2*θ* = 7.8° with *β* = 1.87° is 4.3 nm while the 2H-MoS_2_ grain size calculated from the 2H (002) peak at 2*θ* = 15.1° with *β* = 1.84° is 4.4 nm. We note that the grain size obtained from the XRD patterns for the MoS_2_ samples are a lot smaller than the particle size observed in the TEM micrograph. This indicates that the smaller MoS_2_ grains that give rise to the broader XRD peaks have combined to form larger aggregates seen in the TEM micrograph. From the above XRD results, we can confirm that our 2H-MoS_2_ sample has pure 2H phase and our 1T/2H-MoS_2_ sample has both 1T phase and 2H phase.

The XPS analysis was also used to estimate the phase compositions in the 1T/2H-MoS_2_ and 2H-MoS_2_ samples (Fig. 3d–g). Deconvolution of the XPS spectra was performed using the XPSPEAK41 package^35^. We observed four peaks assigned to Mo^6+^ 3d_3/2_, Mo^6+^ 3d_5/2_& Mo^4+^ 3d_3/2_, Mo^4+^ 3d_5/2_, and S 2s^7,8,28,29,36^ in the XPS spectra of Mo 3d for both samples. In the spectrum for the 1T/2H-MoS_2_ sample (Fig. 3d), the Mo^4+^ 3d_5/2_, and Mo^4+^ 3d_3/2_ peaks for the 1T-MoS_2_ phase are located at 228.31 and 231.51 eV, while those for the 2H-MoS_2_ phase are at 229.31 and 232.51 eV, respectively. The S 2s peak at 225.61 eV for MoS_2_, as well as the Mo^6+^3d_5/2_ peak at 231.64 eV and the Mo^6+^ 3d_3/2_ peak at 234.84 eV, both representing an MoO_3_ impurity in the MoS_2_ sample, are also present. From the fitted peak area ratios, the 1T/2H-MoS_2_ sample was found to be composed of 60.79% 1T-MoS_2_, 30.45% 2H-MoS_2_, and 8.76% MoO_3_. In the spectrum for the 2H-MoS_2_ sample (Fig. 3f), we can see that the 1T-MoS_2_ peaks have disappeared due to the heating process. Apart from the S 2s peak at 226.41 eV, the 2H-MoS_2_ peaks at 229.27 and 233.41 eV, and the impurity MoO_3_ peaks at 232.27 and 235.41 eV are found in the spectrum. The ratios of 2H-MoS_2_ and MoO_3_ in the sample were estimated to be 90.25% and 9.75%, respectively. We also observed two peaks assigned to S 2p_1/2_ and S 2p_3/2_^7,8,28,29,36^ in the XPS spectra of S 2p for both samples. In the spectrum for the 1T/2H-MoS_2_ sample (Fig. 3e), the S 2p_1/2_, and S 2p_3/2_ peaks for the 1T-MoS_2_ phase are located at 162.19 and 161.06 eV, while those for the 2H-MoS_2_ phase are at 163.19 and 162.06 eV, respectively. From the fitted peak area ratios, the 1T/2H-MoS_2_ sample was found to be composed of 64.57% 1T-MoS_2_ and 35.43% 2H-MoS_2_. In the spectrum for the 2H-MoS_2_ sample (Fig. 3g), the 1T-MoS_2_ peaks have disappeared due to the heating process. The 2H-MoS_2_ S 2p_1/2_ and 2p_3/2_ peaks were observed at 163.15 eV and 162.02 eV, respectively. The XPS analysis confirms the presence of 1T-MoS_2_, 2H-MoS_2_, and MoO_3_ in the 1T/2H-MoS_2_ sample. After heating at 300℃ for 1 h in vacuum, the 1T-MoS_2_ was fully transformed into 2H-MoS_2_.

**Figure 4:**
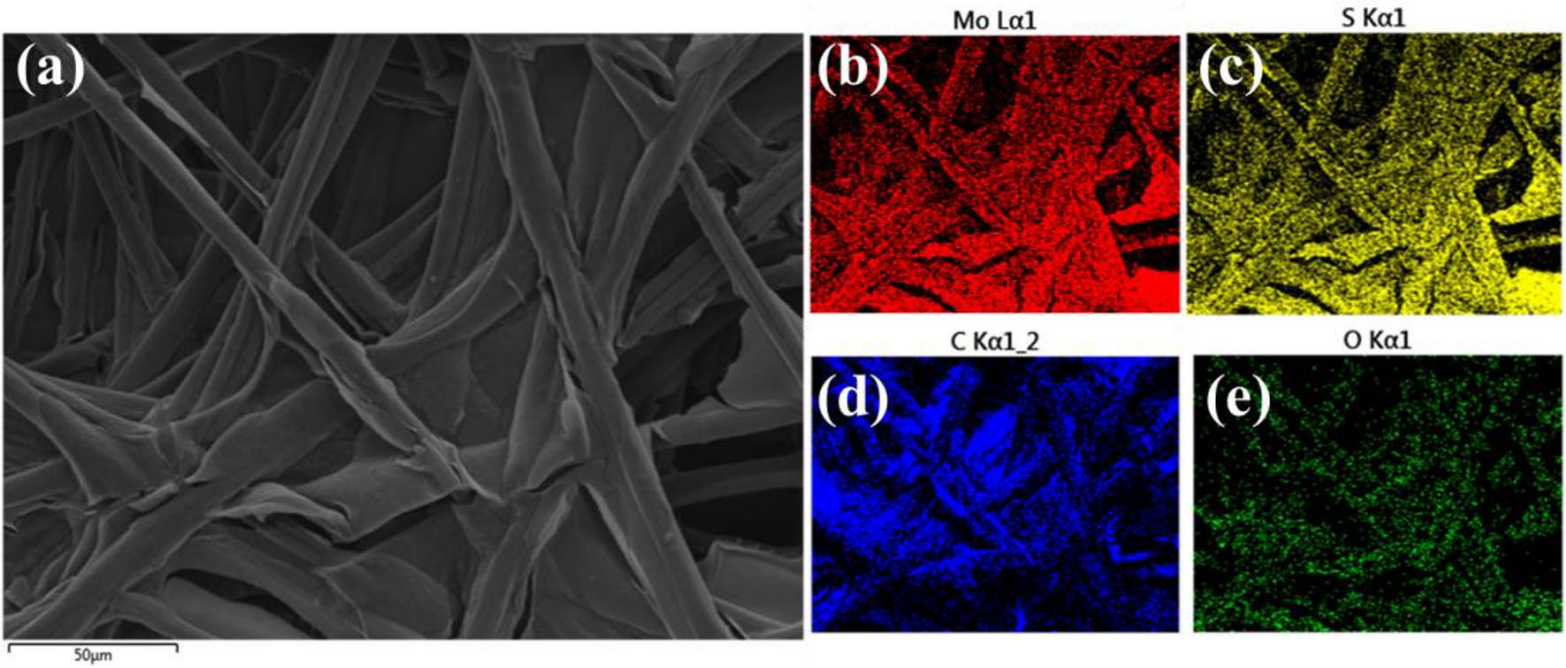
(**a**) SEM image of 1T/2H-MoS_2_/HDGQDs on carbon paper substrate. (**b**—**e**) SEM mapping images of different elements of 1T/2H-MoS_2_/HDGQDs electrode.

The 1T/2H-MoS_2_/HDGQDs composite catalyst system was examined by field emission scanning electron microscopy (FE-SEM). As shown in the SEM micrograph in Fig. 4a, the carbon paper substrate composed of carbon fiber filaments are largely covered by the dried solutes of the 1T/2H-MoS_2_ and HDGQDs solutions. The distribution of each element shown in Fig. 4b-e indicates that the MoS_2_, MnO_3_, and GQDs are distributed along with each other on the carbon paper substrate.

**Figure 5:**
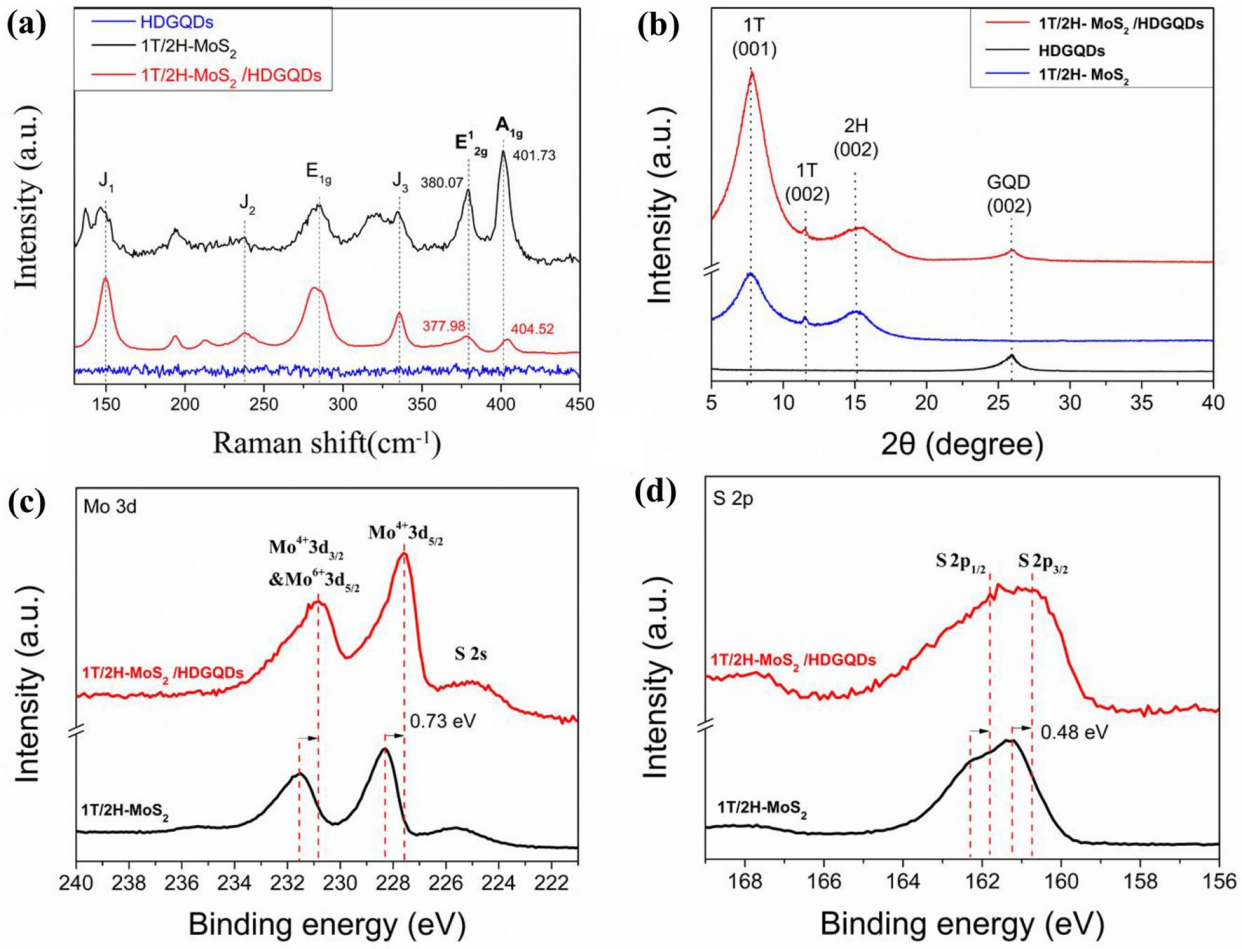
(**a**) The Raman spectra. (**b**) XRD patterns of 1T/2H-MoS_2_, 1T/2H-MoS_2_/HDGQDs, and HDGQDs. (**c**) XPS spectra of Mo 3d for 1T/2H-MoS_2_ and 1T/2H-MoS_2_/HDGQDs. (**d**) XPS spectra of S 2p for 1T/2H-MoS_2_ and 1T/2H-MoS_2_/HDGQDs.

As shown in Fig. 5a, the J_1_, J_2_, J_3_, and E_1g_ peaks specific to the 1T phase of MoS_2_ are present in the Raman spectrum of 1T/2H-MoS_2_/HDGQDs, indicating that the 1T-MoS_2_ phase still exists in 1T/2H-MoS_2_ after HDGQDs doping. However, the A_1g_ peak shifts from 401.73 to 404.52 cm^−1^ and the E^1^_2g_ peak from 380.07 to 377.98 cm^−1^ in the Raman spectrum of 1T/2H-MoS_2_ for the 1T/2H-MoS_2_/HDGQDs compared to that for the 1T/2H-MoS_2_. These peak shifts indicate that the HDGQDs are likely chemically bonded with 1T/2H-MoS_2_ to form a composite catalyst^37,38^. Figure 5b shows the XRD patterns of 1T/2H-MoS_2_, HDGQDs, and 1T/2H-MoS_2_/HDGQDs. The additional peak at 2*θ* = 26.0° for the 1T/2H-MoS_2_/HDGQDs sample, is due to the (002) planes of GQDs. The above XRD results demonstrate that we have successfully synthesized the 1T/2H-MoS_2_/HDGQDs composite materials^39^. As shown in Fig. 5c,d, the peak positions of Mo^6+^ 3d_5/2_& Mo^4+^ 3d_3/2_ and Mo^4+^ 3d_5/2_ were lowered by 0.73 eV and those of S 2p_1/2_ and S 2p_3/2_ by 0.48 eV for 1T/2H-MoS_2_/HDGQDs compared to those for 1T/2H-MoS_2_ in the XPS spectra, indicating charge transfer from HDGQDs to MoS_2_^12^. Such charge transfer also evidences the chemical bonding between HDGQDs and 1T/2H-MoS_2_. The GQDs-to-MoS_2_ charge transfer can improve the efficiency of MoS_2_ in adsorbing protons^12^.

**Figure 6:**
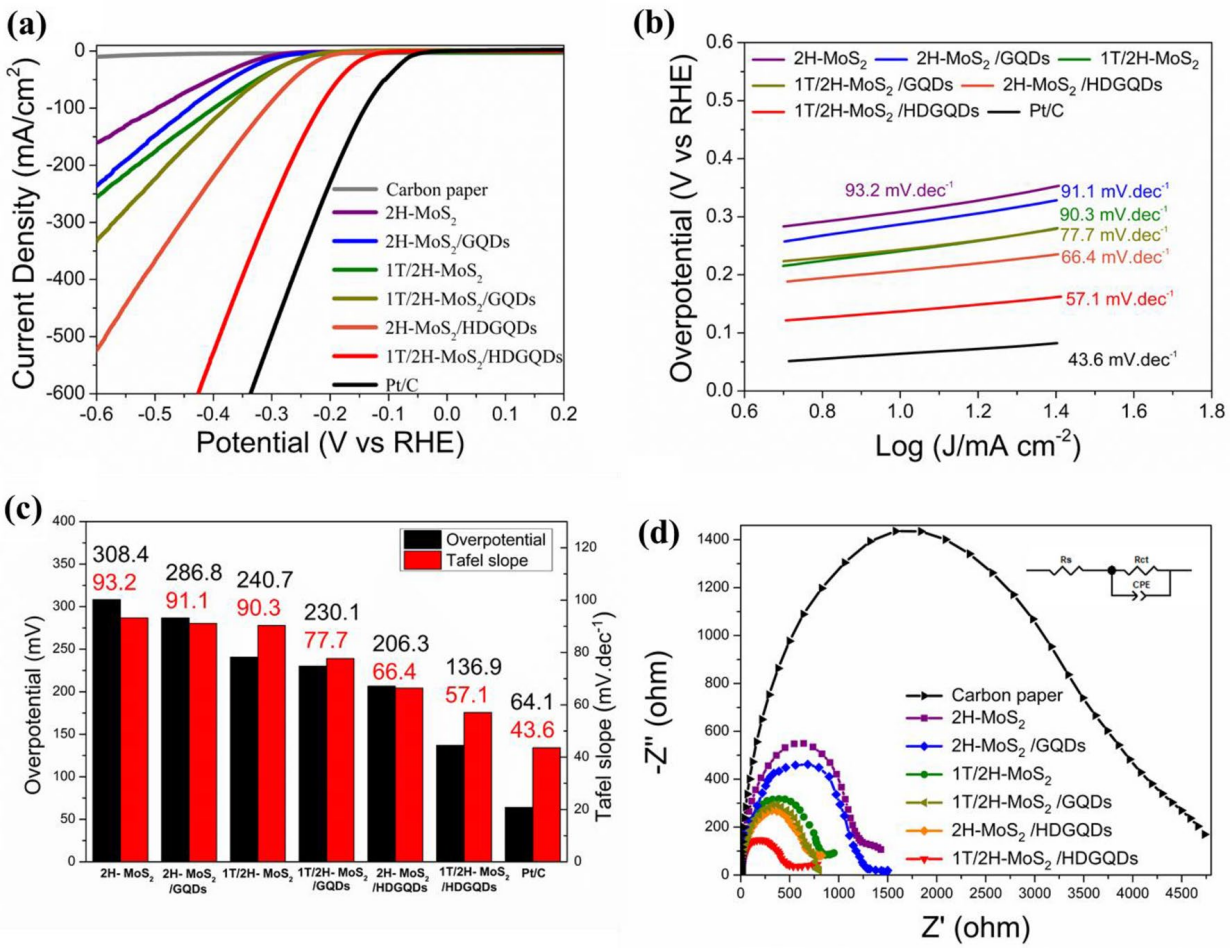
(**a**) Linear sweep voltammetry. (**b**) Tafel plots. (**c**) A duel bar chart to show the overpotentials and Tafel slopes of all samples. (**d**) Electrochemical impedance spectroscopy (EIS).

The LSV curves and Tafel plots for all samples are shown in Fig. 6a,b, respectively. The overpotential and Tafel slope that showcase the electrocatalytic activity for each sample are plotted in Fig. 6c ^40^. As shown in Fig. 6c, the overpotential and Tafel slope for 1T/2H-MoS_2_ are smaller than those for 2H-MoS_2_, indicating better electrocatalytic activity for 1T/2H-MoS_2_ compared to that for 2H-MoS_2_. We can also see that the electrocatalytic activities for both 1T/2H-MoS_2_ and 2H-MoS_2_ were significantly improved when the standard GQDs were incorporated into the samples, while the catalytic activity of the pure HDGQDs and GQDs electrode can be considered negligible (Supplementary Figure S4). Further activity improvement can be achieved by replacing the standard GQDs with the HDGQDs. The best electrocatalytic activity for the MoS_2_/GQDs systems was found in the 1T/2H-MoS_2_/HDGQDs sample, of which the overpotential and Tafel slope of 136.9 mV and 57.1 mV/decade were significantly improved towards the Pt/C values of 64.1 mV and 43.6 mV/decade, respectively.

Figure 6d shows the Nyquist diagrams obtained from the electrochemical impedance spectroscopy (EIS) for all samples, as well as the carbon paper substrate. The corresponding equivalent circuit which consists of solution resistance, *R*_s_, charge-transfer resistance, *R*_ct_, and constant phase element (CPE) is also shown in the inset of Fig. 6d. The EIS parameters (*R*_s_, *R*_ct_, CPE, and the value of the exponent of the CPE (*n*)) obtained from curve-fitting the Nyquist diagrams using the Zview package^41^ are listed in Table 1.^18,42–45^ The charge transfer resistance (*R*_ct_), that originates from the electronic and ionic resistances at the electrode–electrolyte interface, reflects the kinetics of the catalyzed hydrogen evolution reaction^10^.

**Table 1:** The EIS parameters obtained from fitting Nyquist diagrams with the equivalent circuit.

Sample	*R*_s_, Ω	*R*_ct_, Ω	CPE, mF	*n*
Carbon paper	1.5 ± 8.2	3963.0 ± 137.0	0.91 ± 0.03	0.918 ± 0.014
2H-MoS_2_	1.5 ± 1.8	1256.3 ± 32.7	2.99 ± 0.08	0.929 ± 0.011
2H-MoS_2_/GQDs	1.5 ± 1.9	1147.1 ± 34.6	1.26 ± 0.09	0.915 ± 0.038
1T/2H-MoS_2_	1.5 ± 4.1	866.7 ± 22.2	3.37 ± 0.08	0.870 ± 0.006
1T/2H-MoS_2_/GQDs	1.5 ± 0.4	747.1 ± 20.3	4.50 ± 0.20	0.861 ± 0.028
2H-MoS_2_/HDGQDs	1.5 ± 0.6	714.9 ± 23.4	5.57 ± 0.18	0.889 ± 0.015
1T/2H-MoS_2_/HDGQDs	1.5 ± 1.1	537.4 ± 22.6	5.98 ± 0.38	0.808 ± 0.028

Rs is the solution resistance. *R*_ct_ is the charge-transfer resistance. CPE is the constant phase element, and n is the value of the exponent of CPE.

The *R*_ct_ values measured for Carbon paper, 2H-MoS_2_, 2H-MoS_2_/GQDs, 1T/2H-MoS_2_, 1T/2H-MoS_2_/GQDs, 2H-MoS_2_/HDGQDs, and 1T/2H-MoS_2_/HDGQDs are 3963.0 ,1256.3 ,1147.1 ,866.7 ,747.1 ,714.9, and 537.4 Ω, respectively. We can see that, as expected, the metallic-1T-MoS_2_-rich 1T/2H-MoS_2_ sample has a lower *R*_ct_ compared to that of the semiconducting 2H-MoS_2_ sample. When standard GQDs are added to the MoS_2_ samples, both the 2H-MoS_2_ and 1T/2H-MoS_2_ samples show reduced charge-transfer resistance, indicating GQDs have effectively improved the electrode-to-electrolyte charge transfer of the composite catalytic systems. The conductivity can be further improved by replacing the standard GQDs with HDGQDs. We observed the lowest *R*_ct_ of 537.4 Ω in the 1T/2H-MoS_2_/HDGQDs sample, which was dramatically lower than the 2H-MoS_2_ value of 1256.3 Ω.

It has been reported that GQDs can generate abundant defect sites on the basal plane and edge plane of MoS_2_.^10^ These defect sites facilitate electron transfer from GQDs to MoS_2_.^10^ It has also been reported that enhanced GQDs-to-MoS_2_ charge transfer can improve MoS_2_'s efficiency in adsorbing protons.^12^ As demonstrated in our XPS analysis above, we did observe charge transfer from HDGODs to MoS_2_ in our sample. In this work, we use highly defective GQDs to increase the number of defect sites introduced by GQDs, so as to enhance the GQDs-to-MoS_2_ charge transfer, further promoting MoS_2_'s efficiency in adsorbing protons and therefore greatly reducing *R*_ct_ in our catalyzed HER.

According to the above EIS analysis, by selecting 1T/2H-MoS_2_ and HDGQDs to construct our composite catalyst system, we were able to greatly reduce the charge transfer resistance in the catalyzed hydrogen evolution reaction. Furthermore, while 2H-MoS_2_ only has active sites on the edge plane, 1T-MoS_2_ has active sites on the edge plane and basal plane.^3,4,46,47^ When doped with the HDGQDs, 1T/2H-MoS_2_ is likely to have more defects near the active sites. The improved electrode-to-electrolyte charge transfer, larger number of active sites, and additional defects appear to have synergistically led to the greatly enhanced electrocatalytic activity observed in the 1T/2H-MoS_2_/HDGQDs composite catalyst for hydrogen evolution reactions.^3–10,46,47^.

**Figure 7:**
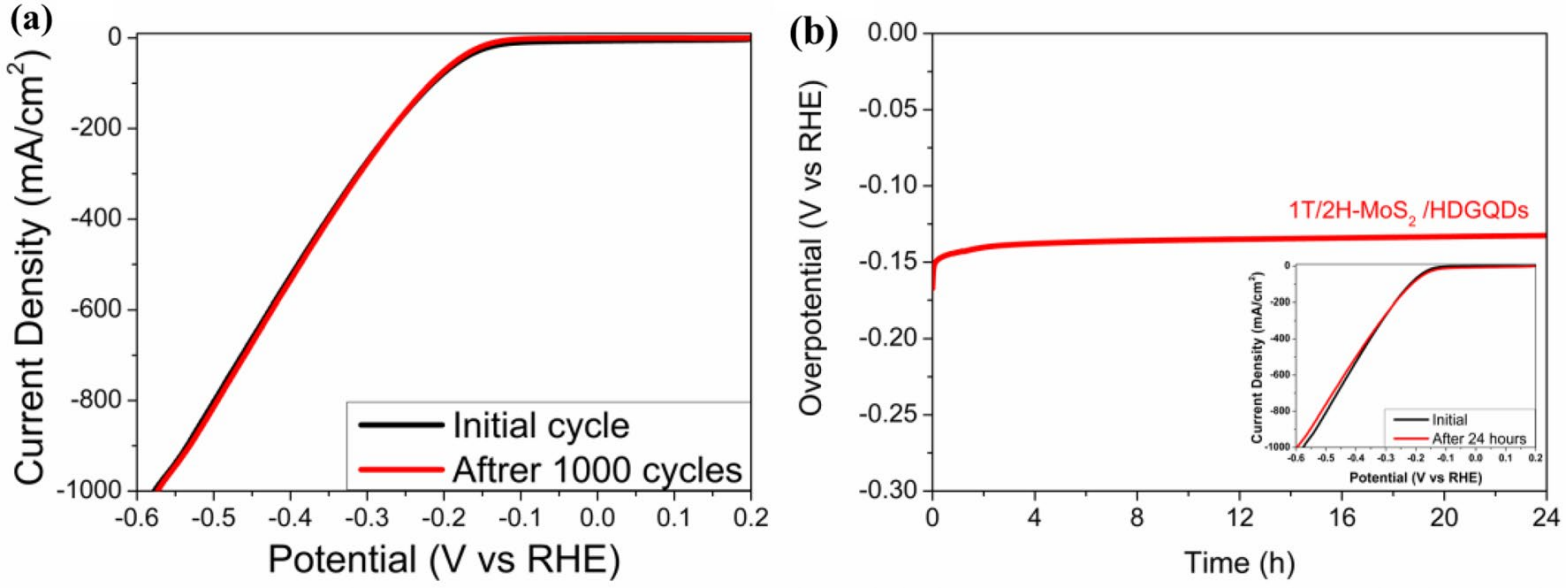
Stability test: (**a**) 1000 LSV cycles and (**b**) The chronopotentiometry curve recorded at a constant cathodic current density of -10 (mA cm^-2^). Inset shows the linear sweep voltammetry polarization curves before and after the 24-h chronopotentiometry.

Finally, a continuous long-term operation of 1000 cycles and a 24-h chronopotentiometry^29,48–50^ were conducted for the 1T/2H-MoS_2_/HDGQDs sample to test its stability. As shown in Fig. 7a, no appreciable difference was found between the LSV curve obtained after 1000 cycles and the initial curve. The chronopotentiometry curve in Fig. 7b shows that the overpotential with a constant current density of 10 mA/cm^2^ was maintained at a stable level for 24 h. As shown in the inset in Fig. 7b, the LSV curves before and after the 24-h chronopotentiometry measurement showed no appreciable difference either. Therefore, our 1T/2H-MoS_2_/HDGQDs composite catalyst appears to be very stable for HER applications.

## Conclusion

We have designed and successfully fabricated an efficient composite catalyst system, consisting of a metallic-1T-MoS_2_-rich 1T/2H-MoS_2_ and a HDGQDs components of particle sizes around 20–40 and 3-5 nm, respectively, for the hydrogen evolution reaction. The 1T/2H-MoS_2_ component was prepared by using an improved process involving a heated mixing of MoS_2_ powder with n-Butyl lithium in an autoclave reactor and a dialysis procedure to significantly expedite the production of 1T-MoS_2_. By using a shortened heating time in pyrolyzing citric acid, we were able to introduce a large number of defects in the GODs product to form HDGQDs, as shown by Raman spectroscopy. While FTIR and XPS data revealed the presence of many functional groups in the HDGQDs that may promote catalytic activity, the XPS data demonstrated that the 1T phase of MoS_2_ is dominant in our 1T/2H-MoS_2_. The FESEM micrographs of the composite catalysts show that both the HDGQDs and 1T/2H-MoS_2_ components distribute along with each other on the carbon paper substrate. The peak shifts in the Raman and XPS spectra of the 1T/2H-MoS_2_/HDGQDs compared to those of the undoped 1T/2H-MoS_2_ indicate successful GQDs doping. Electrochemical measurements show an overpotential of 136.9 mV and Tafel slope of 57.1 mV/decade for the composite catalyst system in HER, demonstrating a large improvement toward the Pt/C values of 64.1 mV and 43.6 mV/decade, respectively. The improved electrode-to-electrolyte charge transfer indicated by EIS data, larger number of active sites in 1T-MoS_2_, and additional defects introduced by HDGQDs doping have synergistically led to the observed electrocatalytic activity enhancement for HER in our composite catalyst system.

### Supplementary Information

Supplementary Information.

## Data Availability

All data generated or analyzed during this study are included in this published article.
