# Supplementary material for: Highly defective graphene quantum dots-doped 1T/2H-MoS2 as an efficient composite catalyst for the hydrogen evolution reaction
Source: Sci Rep. 2023 Sep 13;13:15184. doi: 10.1038/s41598-023-42410-9 (PMC10499812; doi:10.1038/s41598-023-42410-9)
Supplement: Supplementary file 1 — Supplementary Information. [file 41598_2023_42410_MOESM1_ESM.docx]

**Highly defective graphene quantum dots-doped 1T/2H-MoS_2_ as an efficient composite catalyst for the hydrogen evolution reaction**

Sheng-Fu Chen^1^, Tai‑Sing Wu^2,*^ & Yun‑Liang Soo^1,2,*^

^1^*Department of Physics, National Tsing Hua University, Hsinchu, Taiwan*

^2^*National Synchrotron Radiation Research Center, Hsinchu, Taiwan*

**Supplementary Information**

**Optimum loading of the catalyst**





**Supplementary Figure S1.** LSV curves of the 1T/2H-MoS_2_ samples with different MoS_2_ loading.

**
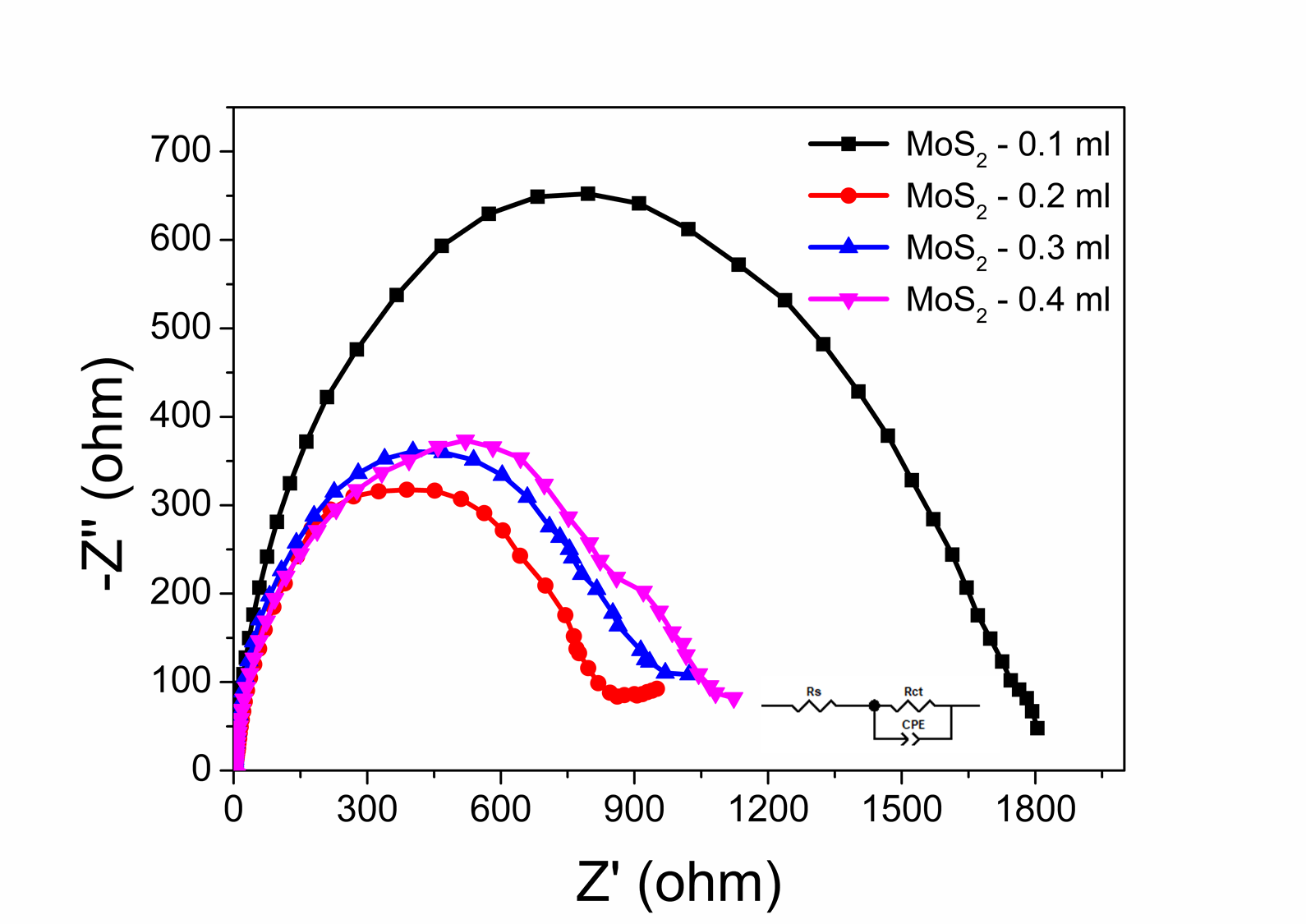
**

**Supplementary Figure S2.** EIS curves of the 1T/2H-MoS_2_ samples with different MoS_2_ loading.

| **Sample** | **R_s_,Ω** | **R_ct_,Ω** | **CPE, mF** | **n** |
| --- | --- | --- | --- | --- |
| MoS_2_ - 0.1 ml | 1.5 ± 1.6 | 1675.0 ± 46.0 | 2.36 ± 0.06 | 0.903 ± 0.011 |
| MoS_2_ - 0.2 ml | 1.5 ± 4.1 | 866.7 ± 22.2 | 3.37 ± 0.08 | 0.870 ± 0.006 |
| MoS_2_ - 0.3 ml | 1.5 ± 0.5 | 888.3 ± 26.3 | 6.84 ± 0.14 | 0.925 ± 0.010 |
| MoS_2_ - 0.4 ml | 1.5 ± 0.6 | 1006.0 ± 26.9 | 5.48 ± 0.12 | 0.867 ± 0.010 |

**Supplementary Table S1.** The EIS parameters obtained by fitting Nyquist diagrams using the equivalent circuit.





**Supplementary Figure S3.** LSV curves of the 1T/2H-MoS_2_/HDGQDs samples with different HDGQDs loading.


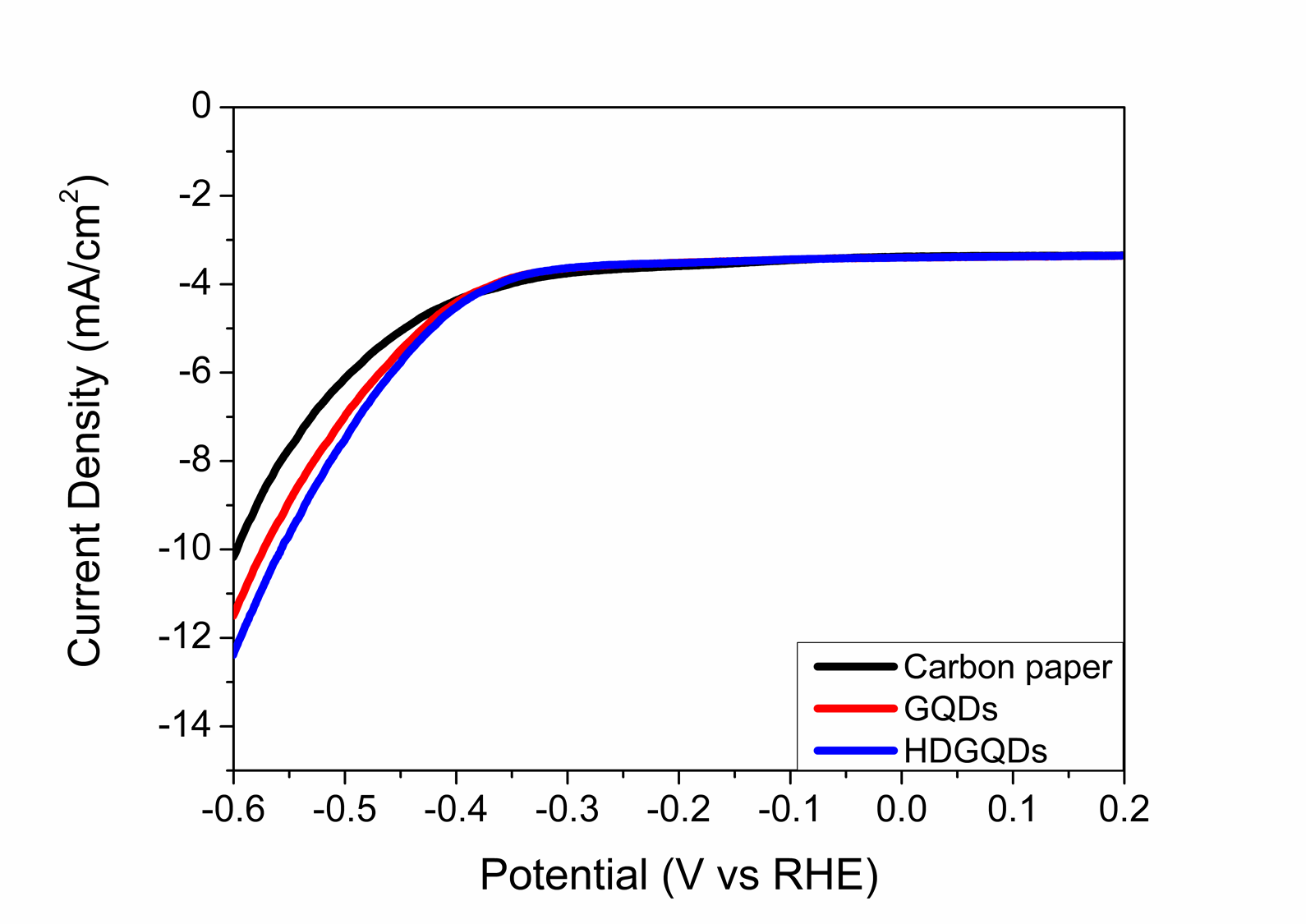


**Supplementary Figure S4.** LSV curves of HDGQDs, GQDs, and Carbon paper.
